# Disabled Students’ perception of the sensory aspects of the learning and social environments within one Higher Education Institution

**DOI:** 10.1177/03080226221126895

**Published:** 2022-10-16

**Authors:** Clodagh Nolan, Jessica K Doyle, Kieran Lewis, Declan Treanor

**Affiliations:** 1 Discipline of Occupational Therapy, Trinity Centre for Health Sciences, Trinity College Dublin, University of Dublin, Dublin, Ireland; 2 Trinity College, University of Dublin, Ireland

**Keywords:** Sensory environment, sensory sensitivities, autistic, disabled students, higher education

## Abstract

**Introduction:**

The environment, both natural and man-made, can influence how we learn and socialise. For some, the environment can be a challenge to overcome. The purpose of this study was to establish a student’s perspective on the sensory aspects of the learning and social environments of a university.

**Methods:**

A survey design based upon Winnie Dunn’s Adult/Adolescent Sensory Profile was developed specifically for this study; 150 disabled students responded to the survey, which was analysed using descriptive statistics and template analysis.

**Results:**

The final template analysis identified 3 main themes with 10 sub-themes, with each sub-theme relating to the research question as well as to the level of explanation: (a) Theme one: *Barriers in the environment* described noise, poor lighting, crowding and lack of visual cues that created difficulties for the respondents to this survey. *The sub-themes* were obstacles to learning in the library, obstacles to learning in lectures, obstacles to learning in exams and ventilation in learning spaces. (b) Theme two: *Reactions to barriers*, included how respondents react to sensory overload and uncertainty in the environment. The *sub-themes* encompassed problems when schedules change regularly, and reactions when overwhelmed in college. (c) Theme three: *Improving the environment* refers to suggestions that respondents made to improving the environment for all students to enable engagement and participation within college. The *sub-themes* compromised of developing a safe space for managing sensory needs, seeking natural elements across campus, seeking awareness, as well as adaptions and strategies for transitions.

**Conclusion:**

Respondents identified how individuals have varied responses to sensory stimuli thereby increasing our understanding. They pointed to a way forward for institutes of higher education to design spaces that are more inclusive by putting forward suggestions for greater use of green space, better furnishings and minimisation of distractions, thereby increasing the health and welfare for all.

## Introduction

Our brains process numerous amounts of information from the sensory environment daily, giving us information on what is happening not only outside our bodies but also what is happening on the inside. We receive and interpret this information through our senses, which enables us to engage in our daily occupations such as eating, dressing, learning, working and socialising. This has been described as sensory processing (Dunn, 1997; Miller and Lane, 2000), which is key to understanding how aspects of the context might support or interfere with participation in everyday occupations (Dunn, 2014). Each individual has a unique way of processing this information and has different sensory preferences (Brown, 2002), reflecting neurodiversity, in the variety of the human brain and the many ways human brains work to perceive, process and interact with the world (Blume, 1998; Silberman, 2018).

For many students, the built environment and context of a college campus can be a barrier to engagement in learning, socialising and performing their activities of daily living, as well as a distracting and overwhelming place to be (Clince et al., 2016; Johnson and Irving, 2008). Over the past 11 years, many Higher Education Institution (HEis) have made significant improvements within their campuses for students with regards to accessibility (AHEAD, 2020), but there remains little focus upon the impact of the ‘sensory environment’ (by this we mean, auditory, visual, tactile, smell, taste, etc.) upon the student population and upon disabled students and those who experience sensory processing differences.

HEIs have an important role to play in the experience of a neurodiverse student population, with a variety of sensory preferences, in providing environments which are conducive to their learning and integration.

In this article, we use identity-first language (‘disabled students’), to reflect the social model of disability, which views disability as central to an individual’s identity (NCDJ, 2021) and furthermore focuses on societal barriers rather than individual medical problems (Dunn and Andrews, 2015). The classification of disabled students includes students with diverse diagnoses such as mental health conditions, Autism, attention deficit hyperactivity disorder (ADHD), physical and sensory impairments and specific learning disabilities. Dunn’s (1997) model of sensory processing, which describes four patterns of sensory processing (low registration, sensation seeking, sensory sensitivity and sensation avoiding), was chosen to inform this research, with its focus upon how individuals function in their environments based upon their sensory preferences (Brown and Dunn, 2010; Dunn, 2007). However, sensory processing has been described in a variety of ways in the literature, reflecting the use of different terminology such as sensory over-responsivity (Miller et al., 2007), sensory processing sensitivity (Aron and Aron, 1997), hyposensitivity and hypersensitivity (Bogdashina, 2016) and sensory defensiveness (Kinnealey et al., 1995).

## Literature review

Johnson and Irving (2008) found that higher education environments can be overwhelming for students with different sensory perceptions which in turn can prevent them from fully engaging in their day-to-day tasks. Equally, Clince et al. (2016) found that neurodivergent students who are Autistic, and having ADHD experienced a range of sensory barriers within higher education environments that impede their integration into the academic and social sphere of college life. Thompson et al. (2019) maintain that Autistic students are likely to encounter many sensory challenges including loud or crowded environments and may require assistance in managing these effectively. Gaines et al. (2016) similarly found that for the Autistic population in their study, the environment can be a help or hindrance to socialising and integrating into college.

Gearhart and Bodie (2012) identified that sensory-processing sensitivity was related to college stress and that those with higher sensory sensitivity reported higher levels of college stress. They found that external stimulation such as loud noise or distracting lights were factors that contributed to stress in sensory-sensitive college students. These findings support the findings by Engel-Yerger and Dunn (2011) that those with sensory sensitivity were more likely to experience anxiety leading to avoidance of activities. Kinnealey et al. (2011) also found that adults with sensory over-responsiveness (heightened sensitivity to or avoidance of sensory stimuli (e.g. loud sounds, bright lights)) had significantly higher scores for anxiety and were perceived to have fewer social supports. They maintain that compatibility with the sensory environment can lead to a better quality of life.

Brown (2002) argues that self-knowledge about how we process information from our environment can enable individuals to establish coping strategies and can help them select and pursue environments that best match their sensory preferences. Kinnealey et al. (1995), Pfeiffer and Kinnealey (2003) and Abernethy (2010) found that adults with sensory defensiveness (the tendency to react with distress or alarm to sensory input) managed sensory sensitivity by using several coping strategies such as avoidance, increasing predictability in tasks, mental preparation, talking through experiences, counteracting and confronting fears and behaviours. Cox et al. (2021) found that students were able to resolve their difficulties if they received personalised adaptations; however, these were not uniformly offered across higher education institutions. Pectu et al. (2021) maintained that students within their first year of entry into college did not use the supports as they did not find them to be specific to their needs.

Madriaga, in a study in 2010, into the lives of students with Asperger’s Syndrome during their transitions into higher education found that most interviewees identified spaces within their universities as being inaccessible such as student unions, pubs and libraries due to their sensory differences and potential for overload within these spaces. Some support staff made suggestions of alternative spaces to reduce sensory overload; however, Madriaga argues that it can have the converse effects of segregating students from a social life and increasing social isolation. Some of the students in her study found themselves in one of two positions either having to face up to their sensory overload issues or face up to isolation. She maintains that students should not be put in such a position and that universities have a duty to take responsibility for eliminating such discrimination. However, for universities and institutes of higher education to implement significant change to environments so that they are inclusive for all, there needs to be a greater understanding of the issues facing those with diverse sensory perceptions and sensory sensitivities.

When creating a range of supports for students within higher education, institutions are often slow to effectively engage with the Autistic community in designing and developing supports (Milton and Bracher, 2013). Van Hees et al. (2015) recommended that supports for Autistic students need to extend beyond academic support and that students should be involved in the planning of these supports. Brown and Dunn (2010) in a study of Autistic children found that behavioural responses to sensory experiences appear to be context related and therefore environmental context is important to examine.

The purpose of this study was to establish a student’s perspective on the sensory aspects of the learning and social environments of a university. Therefore, this study aimed to identify what environmental barriers exist that impact students’ occupational performance from a sensory perspective and to explore possible environmental changes that could be made to learning and social spaces to improve students’ experience and engagement in university life.

## Methodology

This study employed the use of an online survey, which comprised 42 questions, that elicited both qualitative and quantitative data on the students’ perceptions of their sensory environment. Demographic information such as the type of disability, year of study or course was not collected as ethical permission was not granted for the collection of this type of data to protect the students in the HEI of the study. The 42 questions focused on the students’ perceptions of their environment from a sensory perspective based upon Brown and Dunn (2002), to be in line with Dunn’s (1997) Model of Sensory Processing. The survey did not gather information on individual’s sensory profile; instead, the focus was on their view of their learning and social environments. Dunn’s model places a focus upon the environment and therefore it was deemed appropriate to examine the context of a HEI. A survey design was chosen as most of the students had completed all their academic work and were now working away from campus and a survey was considered the best method to reach them. The research was carried out in an Irish HEI of approximately 18,000 undergraduate and postgraduate students, with courses across subject areas in Health Sciences, Arts and Humanities, and Engineering, Maths and Science. All students registered with the disability service at the HEI (*n* = 1629) were sent the survey and invited to participate, of which 150 completed the survey, giving a response rate of 10.9%. Students indicated their consent to participate at the beginning of the survey. This research was granted ethical approval in April 2019 from the Health Science Ethics Committee, Trinity College Dublin.

### Analysis

Quantitative data were analysed using descriptive statistics and the qualitative data were analysed using ‘template analyses’ as described by King (2012). Template Analyses is used to organise and analyse text-based qualitative data thematically. The principal basis of Template Analysis is the construction of a coding template, containing themes identified by the researcher(s) that are significant recurring reflections of the responses and relationships between identified themes found in the data set in relation to the research question, which was ‘*To identify what environmental barriers exist that impact on student’s occupational experience from a sensory perspective*’. Qualitative data were entered into and analysed using Taguette, an online qualitative data analysis tool (Rampin and Rampin, 2021).

Preliminary data coding was carried out by the first and second authors on 20 responses from the 24 qualitative open-ended text responses using provisional a-priori themes based on Brown and Dunn (2002) Model of the four basic patterns of sensory processing (low registration, sensory seeking, sensory sensitivity and sensory avoiding), which were identified in advance as being relevant to the analysis. A-prior themes further were branched into narrower themes nestled under these four broader hierarchical themes connected to each sense as identified by Dunn’s model and connected with the question type with environment type, reaction and whether the response was describing an experience or providing a suggestion.

The second stage involved using the same process to code the entire data set again by the first and second authors. In stage three, there was a review of codes by the research team to ensure expert critique. However, specifying Dunn’s patterns of sensory processing or organising by specific location did not add to the template analysis and was removed. Stage four involved a final revision to the template organised into 3 main themes with 10 sub-themes related by how they connected to the original research question.

## Results

**Figure 1.**
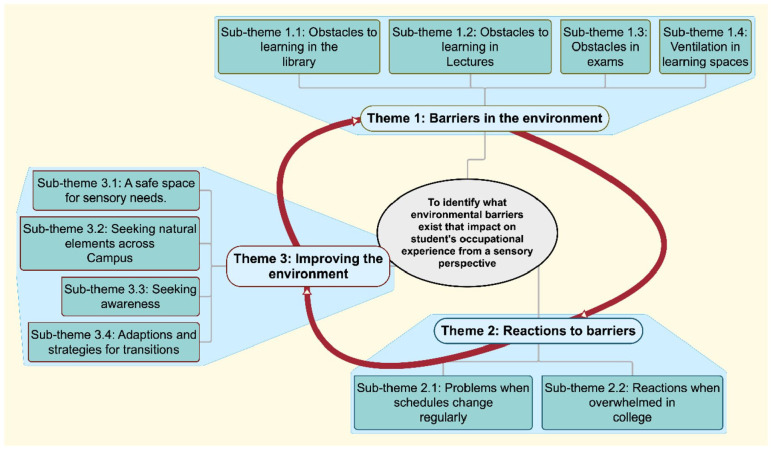
Themes and sub-themes.

The quantitative and qualitative open-ended responses will be discussed together. The final template analysis identified three main themes associated with the learning and social environment with each sub-theme relating to the research question as well as to the level of explanation: (a) Barriers in the environment (four sub-themes), (b) Reactions to barriers (two sub-themes) and (c) Improving the environment (four sub-themes; Figure 1).

### Theme one: Barriers in the environment

Barriers in the environment included noise, poor lighting, too many people in a small space, crowding and lack of visual cues that created difficulties for the respondents in this survey. Sub-themes describe responses that focus on sensory obstacles in specific environments such as sensory distractions in the library that make it hard to concentrate.

*Sub-theme 1.1* focuses on obstacles to learning in the various library settings within the HEI. Noise was a particular issue for students, as one described:

There’s so much going on in the library at all times with people walking around, elevators making noise, etc., that I physically cannot study there. . ..

Another student highlighted the difficulty in finding a study space in the library to suit their sensory preference:

I find it very distracting and lose my concentration and focus easily as some areas are too loud while others are too quiet. . ..

Indeed over 49% (*n* = 73) of the respondents stated that the acoustics were a problem for them in the library. Wayfinding was also highlighted as an obstacle in the library with over 50% (*n* = 75) of students describing signage as poor and having difficulty wayfinding around the libraries.

*Sub-theme 1.2* highlights obstacles to learning in the lecture environment, such as poor lighting, and distracting background noises in lecture halls, as students noted:

Some lighting is too bright. such as lecture halls and can cause headaches. . ..

People talking during lectures make an echo sound and you cannot hear the lecture.

A lack of space was also highlighted, with 64% (*n* = 96) of students describing the desks in learning spaces including lecture halls and libraries to be too close together to individuals in proximity, which then made concentrating in lectures difficult.

*Sub-theme 1.3* explains difficulties faced in exams such as the effects of competing noises and poor acoustics, as a student described:

Can only hear about 20% of what is said. . . echoing on the overhead. Announcements happen often during exams and can go on for a long time, can be disruptive as I lose my train of thought. Sometimes in exams, other exams finish first and they ask everyone to put their pens down. this can be confusing at times. . .

*Sub-theme 1.4* items describe general issues with ventilation and temperature as noted by one student:

tutorial/ laboratories and seminar temperature either very warm or cold due to no fresh air/air conditioning.

Other students described how:

Stuffy air makes me sleepy

Some rooms are so stuffy with no windows it can be hard to concentrate.

Over 55% (*n* = 82) of the respondents maintained that poor ventilation was a problem for them with inconsistent temperatures throughout buildings and lack of fresh air making it difficult to concentrate.

### Theme two: Reaction to barriers

This theme describes how respondents reacted to sensory overload and uncertainty in the environment.

*Sub-theme 2.1* highlights the effect of uncertainty when there are schedule changes and how this affects planning and needing time to adapt to change as is seen in responses such as described by several students:

I forget my timetable has changed and it takes time to adapt.,

Yes, it stresses me out and throws my schedule off

Yes, I get very uncomfortable when I get stuck in the crowds between room changes.

*Sub-theme 2.2* describes items relating to what happens to students becoming overwhelmed and the lack of a safe space on campus, with many retreating to the bathroom, going home or leaving campus. Indeed over 68% (*n* = 102) of respondents stated that there was no quiet space for them to retreat to when feeling overwhelmed. Responses included:

I go home, or I hide in the bathrooms till I am ready to go home. I’m unaware of any safe spaces.

I usually go for a walk or go to a quiet place like a church or a quiet cafe to relax.

I suffer through it – but it massively impacts my productivity, ability to concentrate, stress levels, mood, and mental wellbeing. If it’s very bad, I’ll try and find a safe space (e.g., a bathroom stall). If it’s extremely bad, I’ll go home.

### Theme three: Improving the environment

**Figure 2.**
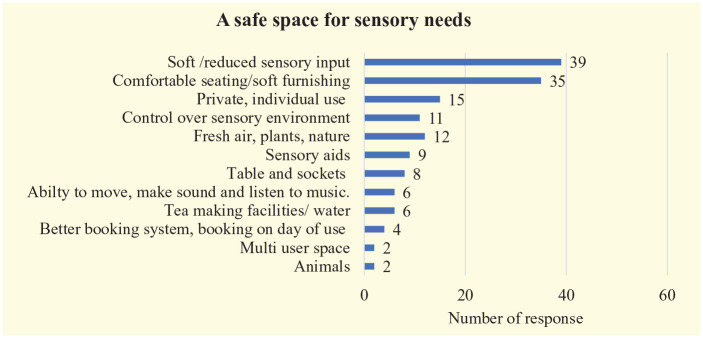
A safe space for sensory needs.

Many of the respondents made suggestions as to how the environment could be improved for all, to enable more engagement and participation within college and give students more control as well as allowing them safe spaces to be themselves and to manage their stress (Figure 2). Students suggested more green areas to be made available as well as improved awareness amongst academic staff of student needs.

*Sub-theme 3.1* highlights the elements people would like to see in a space to manage sensory needs. Items include a mix of different elements with individual control as described in such responses as

I think it will vary greatly depending on time of sensory overload. An inviting minimalist space with greenery.

having a place whre it was acceptable to stim, so move arms about or pace or hum quietly.

Personally, a space that is quiet, dark, and has sensory objects (like fidget toys, weighted objects) would be helpful.

it can be a multiuser space but the rules are it is a quiet, sensory safe haven where one can sit and read or meditate or just try to relax or sleep or rest etc

93% (*n* = 140) of the respondents stated they would use a quiet space if it was available to them. Items also included description of how needing a safe space cannot be planned and booking systems need to account for this, as explained in responses such as:

I cannot know in advance if or when I am going to need the respite room, when I need it I need it now and need it in the same building I am in

. . .Would be good to also have an emergency space that could be used without foresight.

*Sub-theme 3.2* describes items seeking more natural light, nature and plants across campus, both indoors and out as one student described:

The only space I can study is in the library by the windows where I am between windows and books. Small space with natural light is what works best for me.

Other students highlighted:

Soft furnishings, carpet flooring, natural light/non-fluorescent lights. Soft/non reflective surfaces

‘Art on the walls, warm low-level lighting some areas of the library have too harsh lighting, or not enough natural/warm ambient light.’. Another response for a suggestion for study spaces included ‘. more plants’.

*Sub-theme 3.3* items reflect students’ desire for more general awareness across the campus of different sensory preferences and sensory perceptions, as one student noted:

Lecturers need to stop using projectors where they write down their lectures as their primary method of presenting lectures. Esp. when no alternatives are given. . .

Other responses focused on creating this awareness through more effective zoning of learning spaces:

Make more signage showing it is a noise-free zone and better management of spaces

What spaces are quiet/loud and when (all the time or varying?) could be decided on to be made clear, probably with obnoxiously large signs. Modular options for different learning styles/tolerances.

Lastly, *sub-theme 3.4* highlights items requesting more signage and maps to help navigate sensory elements of the environment and in particular transitions between areas, in responses such as:

Noise and visual maps moving from one part of the library to another

Bigger signs with clearer text which are displayed more frequently in the library not just at the entrances.

Other students requested the use for more visuals and colour coding, as one student described:

Colour coded or themed areas rather than just written signs

## Discussion and implications

The findings of this study highlight the importance of an inclusive sensory design and a friendly environment for the academic and social development of the neurodiverse student population, which would enable students to engage and participate in their valued occupation of being a student. In line with other studies (Cox et al., 2021; Krieger et al., 2018; Thompson et al., 2019; Van Hees 2015) this study found that students faced many challenges within the environment that were not conducive to studying, learning or connecting socially with the college community.

Van Hees et al. (2015), Thompson et al. (2019), Cox et al. (2021), Pectu et al. (2021) found that neurodivergent students faced many challenges in their day-to-day experiences within a HEI. This study similarly found that disabled students faced many challenges within the environment, but the challenges were perceived as acting as barriers to engagement and as obstacles that needed to be overcome. The challenges may include a mismatch between environmental design and the ability to process sensory information.

Kinnealey et al. (1995), Anderson et al. (2020) and Ennals et al. (2020) maintain that individuals with sensory sensitivities could develop coping strategies including increasing sensory self-awareness, learning strategies for coping and using academic accommodation for support as a means of managing the sensory environment. Thompson et al. (2019) call upon universities to employ individualised and strengths-based approaches to supporting neurodivergent students in managing their environments. However, none of these studies state how the higher education institutions themselves could provide a more inclusive and accommodating environment or indeed how the environment might change to accommodate students with sensory differences. Cox et al.’s, (2021) study highlighted that HEIs have yet to create environments that do not place the burden upon students when seeking out personal adaptations. More needs to be done to make these education institutions inclusive for all.

Wayfinding with clearly defined access, entries and exits enables one to orient themselves in space (Gaines et al., 2016). Clear signage was identified as an issue for respondents within this study for them to navigate the built environment. People have been found to respond more appropriately when their environments support their needs for meaningful and comprehensive information (Kaplan et al., 2008a). By providing better signage it can allow for better processing of information, and decision making, thus provide a sense of agency and control over one’s environment (Kaplan et al., 2008b).

This study has investigated the disabled students’ perceptions of college environments from a sensory perspective so that any changes made to the learning and social environment would be inclusive of the student voice and meet their needs. Although the students described the challenges under Dunn’s model, it soon became clear that the challenges identified mirrored those of previous studies, but how individuals reacted to those barriers differed with a high proportion of students leaving college or going to the bathroom when overwhelmed. Breakey (2006) calls for HEIs to provide alternative spaces for Autistic students to limit the risk of sensory overload and enable students with sensory sensitivities to manage the environment. Madriaga (2010) refutes this argument stating that it leads to ableism and social segregation. Having to exclude oneself from certain university spaces due to institutional misrecognition of one’s neurodivergence or disability is not reflective of inclusive practice or indeed best practice. She calls for further exploration and discussion around everyday geographies of young people with sensory differences in order to ensure that they do not become isolated. Within this study, students themselves identified the need for a safe space ‘to be themselves’, and to enable them to manage their sensory needs. However, they added that these spaces could be spaces across the campus with natural lighting and plants indoors and outdoors, which could be open to all students. Comfortable seating, plants, controllability over sensory input, fresh air, sensory aids and a multiuser space is what the respondents within this study identified. Like Madriaga (2010), they called for inclusive spaces to be designed for everyone. The implications of these findings mean that we, as occupational therapists, not only need to be aware of different sensory preferences within our client group – in this case, students – but also be cognisant of the sensory environment and the impact it can have on learning, social integration and inclusion for all.

## Practice implications

**Figure 3.**
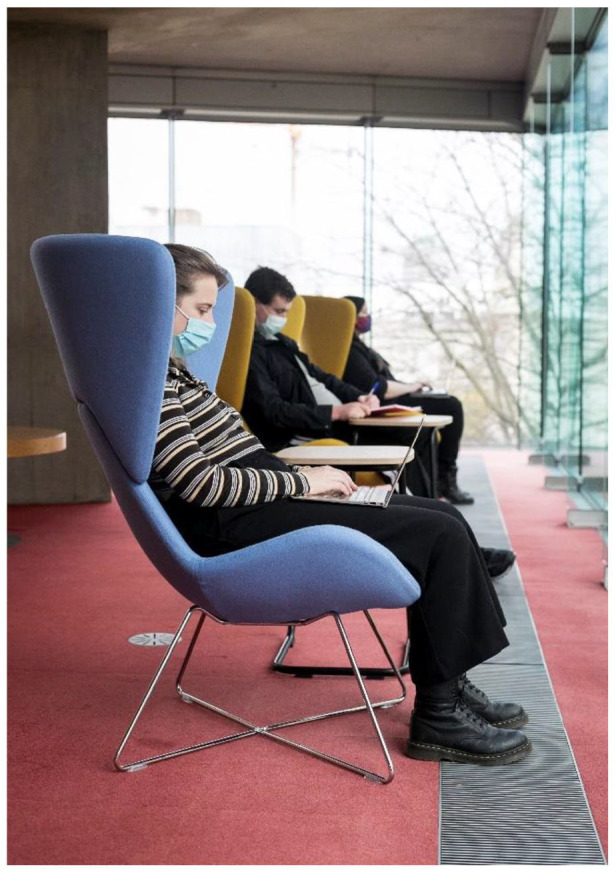
Enclosed seating with the library with views of natural spaces on campus.

**Figure 4.**
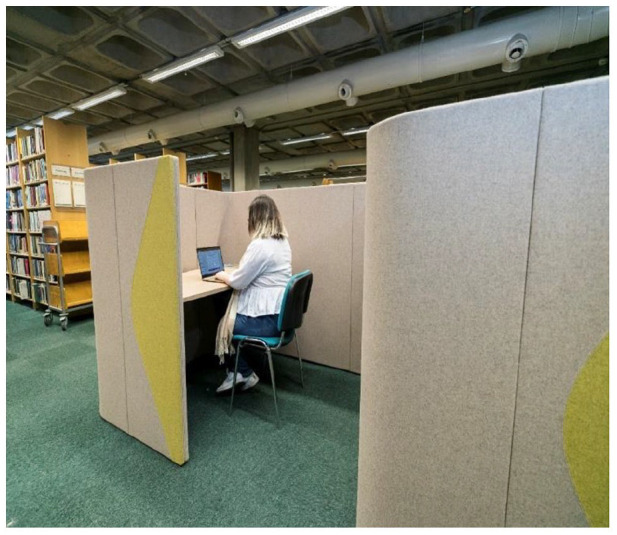
Enclosed study pods to reduce visual distractions.

The outcomes from this study were fed back to the Disability Service in the HEI. This has informed a long-term project, which aims to make the HEI more inclusive by increasing sensory awareness and creating a variety of different sensory environments such as study spaces within the libraries, informal student spaces, mapping of college campus sensory spaces, as well as individual sensory rooms (Figures 3 and 4) to meet the sensory needs of all students and staff across the various HEI campuses. Partnerships have been established between the Disability Service, the Academic Discipline of Occupational Therapy within the HEI, the Library, Disabled Students, the Students Union as well as numerous other areas within the HEI. Funding from a national fund for students with disabilities has not only resulted in significant changes to the campus environment at present, but also to the design of spaces going forward. Below are examples of some of the spaces which have been designed to be more inclusive for all (Figures 3 and 4).

## Limitations

As this study was limited to one HEI using a survey design, it may not be generalisable to other institutions. Disabled students were the target population for the study and therefore this limits the inclusion of all students within higher education whose experiences may differ. Equally, as we were unable to identify specific disability groups, years or academic courses, this limited the analysis of the data; future studies could focus upon specific categories of disability. However, the findings of this study were in line with the findings of other studies and point to important information for the development of more inclusive college campuses for all to live, learn and socialise. It would be important to reach a broader audience of students over several universities to examine if their experiences of the sensory environment are similar or different. Interviews may yield more in-depth results and further research is vital to gain a better understanding of how the sensory environment can affect the student population overall.

## Conclusion

Respondents within this study have pointed to a way forward for universities and institutes of higher education to design spaces that are more inclusive. They identified barriers like others before them did and increased our understanding of how individuals have varied responses to sensory stimuli. They add to the body of knowledge by calling for a reduction in ableism and put forward suggestions for greater use of green space, better furnishings, minimisation of distractions, thereby increasing the health and welfare of all.

## Supplemental Material

sj-docx-1-bjo-10.1177_03080226221126895 – Supplemental material for Disabled Students’ perception of the sensory aspects of the learning and social environments within one Higher Education Institution Supplemental material, sj-docx-1-bjo-10.1177_03080226221126895 for Disabled Students’ perception of the sensory aspects of the learning and social environments within one Higher Education Institution by Clodagh Nolan, Jessica K Doyle, Kieran Lewis and Declan Treanor in British Journal of Occupational Therapy
