# Supplementary material for: Disabled Students’ perception of the sensory aspects of the learning and social environments within one Higher Education Institution
Source: Br J Occup Ther. 2022 Oct 16;86(5):367–75. doi: 10.1177/03080226221126895 (PMC12033423; doi:10.1177/03080226221126895)
Supplement: sj-docx-1-bjo-10.1177_03080226221126895 – Supplemental material for Disabled Students’ perception of the sensory aspects of the learning and social environments within one Higher Education Institution [file sj-docx-1-bjo-10.1177_03080226221126895.docx]

**Environmental Sensory Audit of Learning Spaces:**

**Auditory:**

1. Can you follow the conversation in group settings e.g. tutorials, group work?
2. Can you hear what is being said in public spaces (e.g. libraries, lecture halls)
3. Do you need information to be given in an alternative format?
4. Do you have difficulty with the acoustics (e.g. noises, echoes, humming) in the **library**? If yes please explain
5. If Yes on a scale of 1-5 (5 being the most difficulty and 1 the least) how would you describe this difficulty 1 2 3 4 5
6. Do you have difficulty with the acoustics in rooms where your **lectures** take place? If yes please explain
7. If Yes on a scale of 1-5 (5 being the most difficulty and 1 the least) how would you describe this difficulty 1 2 3 4 5
8. Do you have difficulty with the acoustics in your **exam venue**? If yes please explain and state venue type
9. If Yes on a scale of 1-5 (5 being the most difficulty and 1 the least) how would you describe this difficulty 1 2 3 4 5
10. Are there specific noises that enable you to concentrate, if so please explain?
11. Are there any environmental changes or adaptations that could be made to help you manage such difficulties within these spaces?

**Movement:**

1. Do you think the layout of the library is easy to navigate, including finding books?
2. Is the signage (visual cues) clear to you when moving from one section to the next (e.g. study space/quiet space to public area)?
3. What would help with this transition e.g. to avoid sensory overload?
4. Are the stairwells/lifts easy for you to navigate?
5. Do you think the layout of learning spaces is conducive to learning for you? Please explain
6. Is the purpose of different areas in the library easy to identify?
7. Is there space for you to move without disturbing others if needed?
8. Is there a space for you to manage your sensory needs throughout the day if needed? If No please explain.

**Activity Level**:

1. Is it a problem for you if your schedule changes regularly, i.e. rooms, time changes etc. If Yes please explain.
2. What happens to you if you become overwhelmed in college e.g. do you go home, do you miss class, do you find a safe space? Please explain.
3. Is there a quiet space you can access easily if feeling over-whelmed by the sensory environment?
4. Would you use such a space if it were available within the library?
5. What would you like this space to include?

**Visual:**

1. Do you have difficulty with the lighting or décor in the **library**? If yes please explain
2. Do you have any difficulties with the study areas in the library? Please explain
3. Are there any environmental changes or adaptations that could be made to help you manage difficulties within the study spaces within the library?
4. Which library do you mostly study in:
5. Are there any obstacles that you need to navigate when accessing learning spaces (lecture halls, laboratories etc.)
6. Do you use the furniture within the corridors, common spaces?
7. If so, on a scale of 1-5 (5 being the most difficulty and 1 the least) how would you describe their usefulness 1 2 3 4 5
8. Do you find the corridors and common spaces difficult to navigate at certain times? If yes please explain.
9. Do you have difficulty with the lighting or decor in rooms where your **lectures** take place? If yes please explain
10. Do you have difficulty with the lighting or decor in your **exam venue**? If yes please explain and state venue type
11. Do you use a low distraction venue for exams?
12. If yes, do you have any feedback to provide on these venues?

**Smell**

1. Are there smells in learning spaces that you find overwhelming? Please explain.

**Touch**

1. Are the desks in a classroom/lecture/library in too close a proximity to each other to be comfortable for you?
2. Is the temperature of the classroom/lecture hall/library conducive to learning?
3. Is the ventilation adequate within the lecture/ learning spaces/library? If No please explain.
4. Do you feel your personal space is being invaded within **lectures or learning spaces**? If yes please explain.
5. Do you feel your personal space is being invaded within **exam venues**? If yes please explain.
